# Allograft nephrectomy vs. no nephrectomy for failed renal transplants

**DOI:** 10.3389/fneph.2023.1169181

**Published:** 2023-07-20

**Authors:** Michelle McDonald

**Affiliations:** ^1^ Department of Surgery, University of Texas Southwestern Medical Center, Dallas, TX, United States; ^2^ Department of Urology, University of Texas Southwestern Medical Center, Dallas, TX, United States

**Keywords:** allograft nephrectomy, kidney transplant, immunosuppression, allograft intolerance, failure

## Abstract

The role of allograft nephrectomy (AN) in failed renal transplants is a topic of debate, owing to controversial results reported in the literature and the fact that most of the studies are limited by a retrospective design and small numbers of participants. Allograft nephrectomy is most likely of benefit in the patient with recurrent allograft intolerance syndrome (AIS) following pulse steroids. Immunosuppression weaning in the presence of clinical signs related to a chronic inflammatory state is also reasonable grounds to pursue AN. Studies are mainly inconclusive but suggest that AN has no overall benefit for allograft survival after retransplant. This topic is still of interest in the transplant field and is particularly relevant for patients who are likely to require retransplantation within their lifetime. Further assessment is needed in the form of randomized controlled trials that control for various AN indications and immunosuppression regimens, and have clearly defined survival outcomes.

## Introduction

The number of patients returning to dialysis after kidney transplant is steadily rising because of the overall increase in the number of kidney transplants (1).

The 1-month mortality risk in patients with allograft failure transitioning back to dialysis is nearly seven times that of transplant-naive counterparts (2), and is much higher in the first year than in subsequent years (3). Less than half of kidney transplant failure patients are still alive 10 years after returning to dialysis (4). Retransplant is strongly associated with a survival benefit over dialysis (5), but is only being realized in a small number of patients in the USA. Accordingly, efforts to improve survival rates among those patients with allograft failure and to increase retransplantation rates are of paramount importance.

The role of allograft nephrectomy (AN) in a failed renal transplant patient’s journey is not fully understood. This is closely related to the fact that management of immunosuppression during graft failure is highly variable and the indication for AN is inconsistently reported in the literature. Early vascular thrombosis and allograft intolerance syndrome (AIS) are widely accepted indications for AN, but the utility of AN in an asymptomatic patient is controversial.

To assess for benefit or harm from AN, it must be clear what outcome is being measured—symptom mitigation, allosensitization, patient survival on dialysis, or time to and survival following retransplant.

### Indications for allograft nephrectomy

Allograft nephrectomy is performed at vastly variable rates, reported as 20%–80% of retransplant patients in one recent systematic review (6), and, as noted above, the indication is inconsistently reported. Early graft failure has been defined as any allograft loss in the first year post transplantation (7, 8), and AN is performed in individuals with early graft loss at twice the rate of AN in those who develop later graft loss (8).

Chowaniec et al. found the most commonly reported reason for AN to be AIS (at nearly 50%) (9). Early renal vascular thrombosis is also an accepted indication for AN, given the potential increased risk for allograft rupture and hemorrhage (10). Allograft nephrectomy may also be performed for bleeding, primary non-function, persistent infection, or malignancy, or to create space for retransplant.

### Potential benefits of allograft nephrectomy

#### Immunosuppression withdrawal

Low-dose immunosuppression is frequently maintained while the failed graft is in place to prevent sensitization and to decrease the risk of AIS; however, management strategies vary widely.

Immunosuppression treatments are well known to be associated with infection, increased cardiovascular morbidity, and mortality, which are risks for those who are continued on immunosuppression after graft failure (11–13). An older retrospective cohort study of 197 patients demonstrated that patients who remained on immunosuppression after returning to dialysis had 3.4 times the risk of infection (95% CI 2.5–4.5 times) and a similar increase in mortality (OR 3.4, 95% CI 1.8–6.3), compared with patients who were off immunosuppression (11). Similarly, Smak Gregoor et al. showed a greater risk of infection and death in patients who remained on immunosuppression early after transplant failure (14).

Allograft nephrectomy theoretically offers the potential to mitigate these risks, but there is minimal data to support this. One study from the Australia and New Zealand Dialysis and Transplantation Registry demonstrated risk reversal for infection-related malignancies, including non-Hodgkin’s lymphoma [Epstein–Barr virus (EBV)] and Kaposi’s sarcoma [herpes simplex virus (HSV) type 8], after immunosuppression cessation (15). Many studies do not include a protocol for immunosuppression weaning after transplant failure; therefore, it is hard to draw conclusions on the potential relationship between immunosuppression weaning and the emergence of clinical symptoms, or on the subsequent benefit from AN. A 2019 survey from the American Society of Transplantation (AST) “Kidney Recipients with Allograft Failure, Transition of Care” (KRAFT) working group found that the majority of providers withdraw the antimetabolite first, but one-quarter of respondents reported to having no unified protocol (16).

#### Chronic inflammatory state and allograft intolerance syndrome

A retained failed kidney transplant is often thought to induce a systemic chronic inflammatory state. Patients returning to dialysis may exhibit worse anemia and hypoalbuminemia, have worse C-reactive protein (CRP) levels, and have a poorer erythrocyte sedimentation rate (ESR) than non-transplant dialysis patients with an associated increase in cardiovascular risk, morbidity, and mortality (17, 18). Amelioration of both the clinical and laboratory parameters of a chronic inflammatory state has been observed following AN (18). Furthermore, AN has been associated with improved mortality, which authors have attributed to avoidance of a chronic inflammatory state (17). However, this may represent selection bias and has not been substantiated. Even still, in patients with clinical signs of a chronic inflammatory state despite no obvious symptoms, AN may be considered (19, 20).

A chronic inflammatory state due to immunological intolerance of the failed allograft, along with clinical symptoms, is referred to as AIS (20). In some studies, AIS has been reported in up to 30%–50% of patients within 1 year of allograft failure and dialysis initiation, regardless of the immunosuppression withdrawal protocol used (19, 21). Presentation of AIS is typically within 1 year after transplant failure and common clinical findings include allograft pain, palpable enlargement of the allograft, gross hematuria, and fever. Less common signs of AIS are malaise, weight loss, hematological derangements (thrombocytopenia and resistant anemia), and elevated inflammatory markers (i.e., levels of CRP and ESR) (16, 21, 22).

Some patients may benefit from a pulse of intravenous steroids with maintenance doses of calcineurin inhibitors (CNIs) and oral steroids at moderate doses (16). For AIS patients on dialysis, the KRAFT working group recommends a pulse of steroids followed by a slow taper over 6 months, a CNI trough of 4–6 ng/mL, and a reduction in antimetabolites of 50% for 4–6 weeks. AIS refractory to steriod treatment is the most common indication for nephrectomy and symptoms tend to resolve after AN (22); however, the timing and urgency requires judgment (19). The optimal dose, duration, and number of cycles of steroid treatment prior to surgical intervention is unclear (16).

### Potential harms from allograft nephrectomy

#### Allosensitization

Many patients with failed transplants develop high panel-reactive antibody (PRA) levels only after returning to dialysis therapy. One commonly reported disadvantage of AN and subsequent immunosuppression withdrawal is related to the formation of anti-human leukocyte antigen (HLA) antibodies and the activation of the immune system. This is an important consideration due to the potential for prolonged wait times for subsequent retransplantation, increased acute rejection, and decreased graft survival.

Early AN may minimize allosensitization in patients with graft survival of less than 6 months. Sener et al. demonstrated that patients with early graft loss and AN demonstrated a significant decline in PRA levels at a median follow-up of 4 years, whereas PRA levels remained elevated among those who had AN after late graft loss (23).

There is ample literature suggesting that late AN leads to an increase in class I and class II PRAs, donor-specific antibodies (DSAs), and non-DSAs (19, 24). It is speculated that a retained allograft may serve as an antibody sponge; this theory proposes that preformed anti-HLA antibodies are sequestered in the failed allograft and that AN results in the release of these antibodies into circulation. This was supported by one single-center study that showed a rise in PRA and class I HLA antibodies following AN, whereas maintenance immunosuppression removal resulted in an increase in class II HLA antibodies (25). In addition, anti-HLA antibodies have been found at very high levels in the eluate extracted from excised allografts but not to a similar degree in the serum (26). Similarly, the rapid formation of new DSAs was found following AN in one single-center study, suggesting that the antibodies were preformed (27).

Synergy certainly exists between immunosuppression and AN, contributing to allosensitization. This was seen in a small study by Del Bello et al. measuring patients’ DSA levels using Luminex® technology. Immunosuppression cessation led to increased DSA levels in both AN and non-AN patients, but the increase was more pronounced in AN patients (27). In a recent systematic review assessing the effects of AN on various retransplant end points, half of the included studies found significantly increased PRA levels before retransplant in patients with AN, and the remainder showed no difference. No consistent difference in the class of antibody was found before retransplant using Luminex technology (6).

The conflicting literature about the effects of AN on sensitization is partly due to the fact that AN is often performed after the patient has suffered acute and/or chronic rejection due to weaning of immunosuppression, which results in the sensitizing event occurring prior to AN (16). In addition, the withdrawal of immunosuppression after kidney transplant failure, in the absence of AN, has been shown to predict high sensitization. Augustine et al. analyzed measured the PRA levels of 119 patients with low PRA levels before transplant for 6 to 24 months after graft failure and determined that none of the patients maintained on immunosuppression with CNI underwent AN, whereas 41% of the patients who were weaned off immunosuppression underwent AN for cause. According to multivariate analysis, the weaning of immunosuppression, but not the history of AN, remained significantly correlated to high sensitization (28). A single-center review from Scotland similarly demonstrated a clear association between immunosuppression reduction and rise in sensitization irrespective of AN status and a correlation with decreased chance of retransplant (29). Few studies have reported prophylactic AN prior to immunosuppression weaning, although they suggest it is the events surrounding AN that lead to sensitization rather than the surgery itself (16).

In addition, AN is commonly performed for allograft inflammation and it is not clear whether or not inflammation acts as a “triggering” event to stimulate the production of antibodies. A type of indication bias may occur, and removal of the failed allograft would not necessarily influence the level of pre-existing anti-HLA antibodies. Allograft nephrectomy due to clinical indication has been associated with increased PRA levels when compared with those with elective or no AN (30). Another theory is that AN causes significant tissue damage and elicits an alloimmune response and accelerated cytotoxic antibody formation. Blood transfusions are also likely contribute to the risk of late antibody sensitization because anemia is common after transplant failure.

Compounding the difficulty to draw conclusions on this topic is the heterogeneity in the methods used to identify anti-HLA antibodies in the past 15 years. Some studies include the more recent single-antigen bead technology (Luminex) data to identify DSA, whereas older studies only report PRA level. Allograft nephrectomy and immunosuppression probably act jointly and interdependently in the formation of antibodies.

Overall, the impact of AN on the development of anti-HLA antibodies seems to be neutral or negative (6, 20). Irrespective of the exact mechanism, current evidence shows a trend toward increased anti-HLA antibody formation following AN and consideration of this should be included in decision-making surrounding immunosuppression withdrawal. Recent work has also suggested that a single dose of intravenous immunoglobulin (IVIG) immediately following AN may prevent the formation of DSAs in previously unsensitized patients (31).

#### Surgical morbidity

Owing to the chronic desmoplastic reaction around the allograft and recipient tissues there is a morbidity and mortality risk from AN specific to the surgical procedure itself. Reported mortality from AN varies widely in the literature and is mainly derived from older case studies. Risk is clearly dependent on whether the indication is prophylactic or urgent, with the latter having worse outcomes (19, 20, 32, 33). In most studies, AN-associated morbidity occurred in 13%–26% of recipients, with blood transfusion and infection being the most common morbidities, and mortality occurred in 2%–7% of recipients (34–36), which is higher than the rate reported for kidney transplants (37). There are a number of surgical considerations that contribute to perioperative risk, including graft edema, the size of the graft, the degree of inflammation, and the anemia status of the patient. Preoperative computerized tomography (CT) is frequently used to assess the size and vascular anatomy of the graft, but also to rule out other causes of acute pain and swelling, such as urolithiasis or urinary obstruction. Attention to the preoperative peritoneal dialysis modality is needed, alongside a discussion of potential inadvertent entry into the peritoneum and the possible need to transition to hemodialysis.

The intracapsular AN technique has been associated with shorter operative time and less blood loss than the extracapsular approach (35, 38). This is achieved by making a capsulotomy and using finger dissection to develop the plane between the renal capsule and parenchyma. This approach may allow easier access to the renal hilum for vascular control, but may result in greater donor tissue remaining *in situ.*

Regardless of technique, the preoperative transplant field can have dense scar tissue, obliviating the normal tissue planes and creating a difficult dissection. Tissue can also often be inflamed and edematous due to acute rejection/inflammation or infection and this may lead to poor visualization and contribute to a highly variable degree of intraoperative blood loss and the potential for vascular injury. Confirmation of an easily palpable femoral pulse is performed frequently to ensure the viability of extremity vasculature.

### Alternative surgical option of percutaneous vascular embolization

Embolization of the allograft renal artery has been proposed as a less morbid alternative to AN (36). This is accomplished by injection of ethanol followed by stainless steel coils into the renal artery (36, 39). One systematic review and meta-analysis of 2,232 AN and 189 percutaneous embolization patients concluded that renal artery embolization may result in fewer risks, such as bleeding and infection, and successfully ameliorated AIS in 80% of patients without AN. However, post-embolization syndrome, described as fever, pain, nausea and vomiting, was high at 68% and 20% of patients needed post-embolization AN (36). In large-volume transplant centers where radiology or vascular teams are regularly providing embolization for AIS, percutaneous embolization is a potentially less morbid procedure for some and may be considered in decision-making.

### Effect on retransplantation

There are conflicting results on the impact of AN on the need for future retransplantation due to inconsistent reporting and variable outcome assessment. In a 2010 US study, Ayus et al. reported that patients who underwent AN after transplant failure were twice as likely to receive a second transplant and had improved survival (17). Conversely, a systematic review from 2022 of 15 retrospective cohort studies comprising 5,431 patients did not observe an allograft survival or patient survival benefit after retransplant for patients who had undergone AN, and four studies found worse allograft survivial rates (6). Only one of the 15 studies found that in the patient subgroup with early kidney allograft failure (< 12 months post transplant), AN may be associated with improved retransplant survival (6). It has been argued that if symptomatic immunological responses prompted AN, then this is simply a marker of high immunological risk for repeat transplant failure (19).

Retrospective analysis of AN prior to retransplant in groups with similar PRA levels revealed no significant impact on subsequent outcomes (19). A 2016 meta-analysis of eight retrospective studies comprising 1,008 patients assessed retransplant outcomes based on AN status and found that patients in the AN group had longer time to retransplant and higher rates of PRA > 10% before retransplant, but this did not appear to affect graft and patient survival (24). This is in contrast to a 2018 meta-analysis, which concluded that 3- and 5-year graft survival was significantly lower in the AN cohort than in the no-AN cohort (40).

Conversely, a more recent meta-analysis of 16 studies from 1990 to 2021 reported a significant increase in rates of delayed graft function (DGF), %PRA, acute rejection, and primary non-function in the AN cohort; however, AN prior to retransplant was not associated with worse 5-year graft and patient survival. Despite the increase in %PRA in the AN cohort, time to retransplant demonstrated non-significant differences between the two groups (7). Donation after cardiac death (DCD) rates, kidney donor profile index (KDPI) values, and other donor profile information were not included in the analysis.

Overall, the impact of AN on retransplant outcomes does not seem to offer an advantage in avoiding kidney transplant failure.

## Conclusion

Allograft failure can pose challenges in management to the transplant community. Most studies assessing the role of AN are limited by their retrospective and single-center nature, widely variable follow-up periods, and the absence of the indication for AN or immunosuppression weaning protocol. In addition, many studies are underpowered to assess the effect of AN on patient survival.

Allograft nephrectomy is most likely of benefit in patients with recurrent AIS following a pulse of steroids.

Clinical signs related to a chronic inflammatory state occurring during immunosuppression weaning are reasonable grounds to pursue AN.

Irrespective of the exact mechanism, current evidence shows a trend toward increased anti-HLA antibody formation following AN and consideration of this should be included in tailored decision-making. Studies are mainly inconclusive but suggest no overall benefit from AN for allograft survival after retransplant. The consideration of allosensitization and goal of retransplantation are tempered by the surgical morbidity and mortality rates of AN.

To fully understand the benefit or harm from AN following graft failure, further assessment is needed in the form of randomized controlled trials that control for various AN indications and immunosuppression regimens, and have clearly defined survival outcomes.

## Author contributions

The author confirms being the sole contributor to this work and has approved it for publication.

## References

[1] Hart A Smith JM Skeans MA Gustafson SK Wilk AR Castro S. OPTN/SRTR 2018 annual data report: kidney. Am J Transplant (2020) 20 Suppl s1:20–130. doi: 10.1111/ajt.15672 31898417

[2] Rao PS Schaubel DE Jia X Li S Port FK Saran R. Survival on dialysis post-kidney transplant failure: results from the scientific registry of transplant recipients. Am J Kidney Dis (2007) 49(2):294–300. doi: 10.1053/j.ajkd.2006.11.022 17261432

[3] Kabani R Quinn RR Palmer S Lewin AM Yilmaz S Tibbles LA. Risk of death following kidney allograft failure: a systematic review and meta-analysis of cohort studies. Nephrol Dial Transplant (2014) 29(9):1778–86. doi: 10.1093/ndt/gfu205 24895440

[4] Kaplan B Meier-Kriesche HU. Death after graft loss: an important late study endpoint in kidney transplantation. Am J Transplant (2002) 2(10):970–4. doi: 10.1034/j.1600-6143.2002.21015.x 12482151

[5] Rao PS Schaubel DE Wei G Fenton SS. Evaluating the survival benefit of kidney retransplantation. Transplantation (2006) 82(5):669–74. doi: 10.1097/01.tp.0000235434.13327.11 16969291

[6] Vlachopanos G El Kossi M Aziz D Halawa A. Association of nephrectomy of the failed renal allograft with outcome of the future transplant: a systematic review. Exp Clin Transplant (2022) 20(1):1–11. doi: 10.6002/ect.2021.0133 34775942

[7] Gavriilidis P O'Callaghan JM Hunter J Fernando T Imray C Roy D. Allograft nephrectomy versus nonallograft nephrectomy after failed renal transplantation: a systematic review by updated meta-analysis. Transpl Int (2021) 34(8):1374–85. doi: 10.1111/tri.13901 34062020

[8] Johnston O Rose C Landsberg D Gourlay WA. Gill JS. Nephrectomy after transplant failure: current practice and?outcomes. Am J Transplant (2007) 7(8):1961–7. doi: 10.1111/j.1600-6143.2007.01884.x 17617860

[9] Chowaniec Y Luyckx F Karam G Glemain P Dantal J Rigaud J. Transplant nephrectomy after graft failure: is it so risky? impact on morbidity, mortality and alloimmunization. Int Urol Nephrol (2018) 50(10):1787–93. doi: 10.1007/s11255-018-1960-4 30120679

[10] Azar GJ Zarifian AA Frentz GD Tesi RJ Etheredge EE. Renal allograft rupture: a clinical review. Clin Transplant (1996) 10(6 Pt 2):635–8. 8996757

[11] Gregoor PJ Kramer P Weimar W van Saase JL. Infections after renal allograft failure in patients with or without low-dose maintenance immunosuppression. Transplantation (1997) 63(10):1528–30. doi: 10.1097/00007890-199705270-00028 9175823

[12] Johnston O Zalunardo N Rose C Gill JS. Prevention of sepsis during the transition to dialysis may improve the survival of transplant failure patients. J Am Soc Nephrol (2007) 18(4):1331–7. doi: 10.1681/ASN.2006091017 17314323

[13] Woodside KJ Schirm ZW Noon KA Huml AM Padiyar A Sanchez EQ. Fever, infection, and rejection after kidney transplant failure. Transplantation (2014) 97(6):648–53. doi: 10.1097/01.TP.0000437558.75574.9c 24637864

[14] Smak Gregoor PJ Zietse R van Saase JL op de Hoek CT Jzermans I JN Lavrijssen AT. Immunosuppression should be stopped in patients with renal allograft failure. Clin Transplant (2001) 15(6):397–401. doi: 10.1034/j.1399-0012.2001.150606.x 11737116

[15] van Leeuwen MT Webster AC McCredie MR Stewart JH McDonald SP Amin J. Effect of reduced immunosuppression after kidney transplant failure on risk of cancer: population based retrospective cohort study. BMJ (2010) 340:c570. doi: 10.1136/bmj.c570 20150194 PMC2820609

[16] Lubetzky M Tantisattamo E Molnar MZ Lentine KL Basu A Parsons RF. The failing kidney allograft: a review and recommendations for the care and management of a complex group of patients. Am J Transplant (2021) 21(9):2937–49. doi: 10.1111/ajt.16717 34115439

[17] Ayus JC Achinger SG Lee S Sayegh MH Go AS. Transplant nephrectomy improves survival following a failed?renal allograft. J Am Soc Nephrol (2010) 21(2):374–80. doi: 10.1681/ASN.2009050480 PMC2834539 19875809

[18] Lopez-Gomez JM Perez-Flores I Jofre R Carretero D Rodriguez-Benitez P Villaverde M. Presence of a failed kidney transplant in patients who are on hemodialysis is associated with chronic inflammatory state and erythropoietin resistance. J Am Soc Nephrol (2004) 15(9):2494–501. 10.1097/01.ASN.0000137879.97445.6E 15340000

[19] Pham PT Everly M Faravardeh A Pham PC. Management of patients with a failed kidney transplant: dialysis reinitiation, immunosuppression weaning, and transplantectomy. World J Nephrol (2015) 4(2):148–59. doi: 10.5527/wjn.v4.i2.148 PMC4419125 25949929

[20] Ayus JC Achinger SG. At The peril of dialysis patients: ignoring the failed transplant. Semin Dial (2005) 18(3):180–4. doi: 10.1111/j.1525-139X.2005.18304.x 15934958

[21] Delgado P Diaz F Gonzalez A Sanchez E Gutierrez P Hernandez D. Intolerance syndrome in failed renal allografts: incidence and efficacy of percutaneous embolization. Am J Kidney Dis (2005) 46(2):339–44. doi: 10.1053/j.ajkd.2005.04.024 16112054

[22] Khakhar AK Shahinian VB House AA Muirhead N Hollomby DJ Leckie SH. The impact of allograft nephrectomy on percent panel reactive antibody and clinical outcome. Transplant Proc (2003) 35(2):862–3. doi: 10.1016/S0041-1345(02)04031-9 12644168

[23] Sener A Khakhar AK Nguan CY House AA Jevnikar AM Luke PP. Early but not late allograft nephrectomy reduces allosensitization after transplant failure. Can Urol Assoc J (2011) 5(6):E142–7. doi: 10.5489/cuaj.10032 PMC3235219 21388588

[24] Wang K Xu X Fan M Qianfeng Z. Allograft nephrectomy vs. no-allograft nephrectomy for renal transplantation: a meta-analysis. Clin Transplant (2016) 30(1):33–43. 26458229 10.1111/ctr.12654

[25] Lachmann N Schonemann C El-Awar N Everly M Budde K Terasaki PI. Dynamics and epitope specificity of anti-human leukocyte antibodies following renal allograft nephrectomy. Nephrol Dial Transplant (2016) 31(8):1351–9. doi: 10.1093/ndt/gfw041 27190369

[26] Martin L Guignier F Mousson C Rageot D Justrabo E Rifle G. Detection of donor-specific anti-HLA antibodies with flow cytometry in eluates and sera from renal transplant recipients with chronic allograft nephropathy. Transplantation (2003) 76(2):395–400. doi: 10.1097/01.TP.0000078895.24606.45 12883199

[27] Del Bello A Congy-Jolivet N Sallusto F Guilbeau-Frugier C Cardeau-Desangles I Fort M. Donor-specific antibodies after ceasing immunosuppressive therapy, with or without an allograft nephrectomy. Clin J Am Soc Nephrol (2012) 7(8):1310–9. doi: 10.2215/CJN.00260112 PMC3408115 22626959

[28] Augustine JJ Woodside KJ Padiyar A Sanchez EQ Hricik DE Schulak JA. Independent of nephrectomy, weaning immunosuppression leads to late sensitization after kidney transplant failure. Transplantation (2012) 94(7):738–43. doi: 10.1097/TP.0b013e3182612921 22955228

[29] Nimmo A McIntyre S Turner DM Henderson LK Battle RK. The impact of withdrawal of maintenance immunosuppression and graft nephrectomy on HLA sensitization and calculated chance of future transplant. Transplant Direct (2018) 4(12):e409. doi: 10.1097/TXD.0000000000000848 30584590 PMC6283087

[30] Sumrani N Delaney V Hong JH Daskalakis P Sommer BG. The influence of nephrectomy of the primary allograft on retransplant graft outcome in the cyclosporine era. Transplantation (1992) 53(1):52–5. doi: 10.1097/00007890-199201000-00009 1733085

[31] Matignon M Leibler C Moranne O Salomon L Charron D Lang P. Anti-HLA sensitization after kidney allograft nephrectomy: changes one year post-surgery and beneficial effect of intravenous immunoglobulin. Clin Transplant (2016) 30(6):731–40. doi: 10.1111/ctr.12743 27140447

[32] Sharma DK Pandey AP Nath V Gopalakrishnan G. Allograft nephrectomy–a 16-year experience. Br J Urol (1989) 64(2):122–4. doi: 10.1111/j.1464-410X.1989.tb05969.x 2670045

[33] Alberts VP Minnee RC Bemelman FJ van Donselaar-van der Pant KA Idu MM. Transplant nephrectomy: what are the surgical risks? Ann Transplant (2013) 18:174–81. doi: 10.12659/AOT.883887 23792518

[34] Akoh JA. Transplant nephrectomy. World J Transplant (2011) 1(1):4–12. doi: 10.5500/wjt.v1.i1.4 24175187 PMC3782229

[35] Vavallo A Lucarelli G Bettocchi C Tedeschi M Palazzo S Losappio V. Allograft nephrectomy: what is the best surgical technique? Transplant Proc (2012) 44(7):1922–5. doi: 10.1016/j.transproceed.2012.06.011 22974872

[36] Takase HM Contti MM Nga HS Bravin AM Valiatti MF El-Dib RP. Nephrectomy versus embolization of non-functioning renal graft: a systematic review with a proportional meta-analysis. Ann Transplant (2018) 23:207–17. doi: 10.12659/AOT.907700 PMC6248024 29581414

[37] Wolfe RA Ashby VB Milford EL Ojo AO Ettenger RE Agodoa LY. Comparison of mortality in all patients on dialysis, patients on dialysis awaiting transplantation, and recipients of a first cadaveric transplant. N Engl J Med (1999) 341(23):1725–30. doi: 10.1056/NEJM199912023412303 10580071

[38] Touma NJ Sener A Caumartin Y Warren J Nguan CY Luke PP. Extracapsular versus intracapsular allograft nephrectomy: impact on allosensitization and surgical outcomes. Can Urol Assoc J (2011) 5(1):49–52. doi: 10.5489/cuaj.10016 21470516 PMC3036759

[39] Lorenzo V Contti MM Nga HS Bravin AM Valiatti MF El-Dib RP. Ablation of irreversibly rejected renal allograft by embolization with absolute ethanol: a new clinical application. Am J Kidney Dis (1993) 22(4):592–5. doi: 10.1016/S0272-6386(12)80934-6 8213801

[40] Lin J Wang R Xu Y Chen J. Impact of renal allograft nephrectomy on graft and patient survival following retransplantation: a systematic review and meta-analysis. Nephrol Dial Transplant (2018) 33(4):700–8. doi: 10.1093/ndt/gfx360 29444290

